# Reinforcement Learning (RL)-Based Energy Efficient Resource Allocation for Energy Harvesting-Powered Wireless Body Area Network

**DOI:** 10.3390/s20010044

**Published:** 2019-12-19

**Authors:** Yi-Han Xu, Jing-Wei Xie, Yang-Gang Zhang, Min Hua, Wen Zhou

**Affiliations:** 1 College of Information Science and Technology, Nanjing Forestry University, Nanjing 210037, China; xjw1224@njfu.edu.cn (J.-W.X.); min_hua@njfu.edu.cn (M.H.); wenzhou@ustc.edu (W.Z.); 2 School of Electrical Engineering and Telecommunications, University of New South Wales, Sydney 2052, Australia; 3 School of Information Science and Technology, Fudan University, Shanghai 200433, China; to19210720125@fudan.edu.cn

**Keywords:** energy harvesting, wireless body area networks, reinforcement learning, resource allocation, energy efficient

## Abstract

Wireless body area networks (WBANs) have attracted great attention from both industry and academia as a promising technology for continuous monitoring of physiological signals of the human body. As the sensors in WBANs are typically battery-driven and inconvenient to recharge, an energy efficient resource allocation scheme is essential to prolong the lifetime of the networks, while guaranteeing the rigid requirements of quality of service (QoS) of the WBANs in nature. As a possible alternative solution to address the energy efficiency problem, energy harvesting (EH) technology with the capability of harvesting energy from ambient sources can potentially reduce the dependence on the battery supply. Consequently, in this paper, we investigate the resource allocation problem for EH-powered WBANs (EH-WBANs). Our goal is to maximize the energy efficiency of the EH-WBANs with the joint consideration of transmission mode, relay selection, allocated time slot, transmission power, and the energy constraint of each sensor. In view of the characteristic of the EH-WBANs, we formulate the energy efficiency problem as a discrete-time and finite-state Markov decision process (DFMDP), in which allocation strategy decisions are made by a hub that does not have complete and global network information. Owing to the complexity of the problem, we propose a modified Q-learning (QL) algorithm to obtain the optimal allocation strategy. The numerical results validate the effectiveness of the proposed scheme as well as the low computation complexity of the proposed modified Q-learning (QL) algorithm.

## 1. Introduction

Recent advances in sensors and wireless communication technology have resulted in a promising development of wireless body area networks (WBANs) [1]. The specific application scenario of WBANs is to continuously monitor the vital physiological signals of the human body and transmit the real-time sensory data to the users and doctors without any interruptions in their daily lifestyle to realize smart healthcare in the framework of smart cities [2,3]. Different from the conventional complex and wired healthcare devices, WBANs typically consist of a number of battery-driven, invasive, and/or non-invasive body sensors and one hub (mobile phone or personal digital assistant (PDA)) with the communication function in the form of wireless. The hub normally has rich resources, such as energy supply, processing capability, and buffer storage. In contrast, body sensors are energy-limited owing to the small size. Meanwhile, it is difficult or inconvenient to replace the battery as these body sensors may be implanted in the human body. Therefore, designing an energy efficient resource allocation scheme has great significance to WBANs. A comprehensive survey on the major characteristics, research issues, and challenges in WBANs for patient monitoring from a practical design and implementation perspective was provided in the works of [4,5]. Additionally, several previous works in the literature were proposed to investigate the energy-saving technologies from the aspects of the media access control (MAC) protocol design, power control, and cross-layer resource allocation strategies to make efforts to prolong the lifetime of WBANs [6,7,8,9,10,11,12]. In the work of [7], the authors presented a time division multiple access (TDMA)-based technique to improve WBANs’ reliability and energy efficiency by adaptively synchronizing nodes while tackling channel and buffer status. The simulation results show that the method can improve packet loss and energy consumption. In the work of [9], a reinforcement learning-based sensor access control scheme in WBANs was investigated. The authors considered the signal-to-interference plus noise ratio, transmission priority, battery level, and transmission delay to make a decision on the access time and transmit power. In the work of [10], a multi-hop routing protocol and routing decision strategies in WBANs for medical application were proposed. The authors formulated the optimization problem into Markov decision process (MDP) subjects to various conditions such as battery level, event occurrence, packet transmission rate, and link quality. However, these schemes cannot ensure the WBANs achieve the ultimate goal of ‘uninterrupted work’. Once these batteries are drained, the sensors will be dead. In addition to further improving energy efficiency, energy harvesting is an appealing solution [13,14,15]. Energy harvesting is a technology that enables devices to collect energy from ambient sources [16]. Various types of energy sources can be exploited as energy supplies, for instance, thermal, locomotion, and electromagnetic wave [17,18]. Meanwhile, in the existing literature, several energy harvesting models have also been investigated in the literature [19,20,21]. Therefore, energy harvesting (EH)-powered WBANs (EH-WBANs) have the potential ability to achieve the infinite lifetime and perpetual operation, which is called energy neutral operation (ENO) [22]. However, owing to the fluctuation of ambient energy source and the immaturity of energy conversion technology, the available energy of each body sensor will become a vital factor in the design of resource allocation schemes in EH-WBANs. A comprehensive survey on energy scavengers and their potential utilization in WBANs was given in the work of [23]. In another paper [24], a wireless charged wearable network for monitoring physiological signals of patients was investigated. The authors proposed to cluster the wearable devices to improve the reliability and lifetime of the network. In the work of [25], two scenarios of a point-to-point communication system in WBANs were studied, in which two protocols, called the power splitting protocol and time switching protocol, were proposed to maximize the network throughput.

To the best of our knowledge, the research on resource allocation for EH-WBANs is in its infancy, despite having some pioneering studies [26,27,28,29,30,31,32,33,34,35]. Mohammadi et al. [26] proposed a link adaption mechanism to maximize energy efficiency in Institute of Electrical and Electronics Engineers (IEEE) 802.15.6 impulse radio ultra-wideband (IR-UWB) WBANs. In the work of He et al. [27], the transmission power and source rate of sensors are jointly optimized to provide quality of service (QoS) requirements for WBANs. However, the authors only considered single hop transmission between the sensor and coordinator. Liu et al. [28] proposed a transmission rate allocation scheme to efficiently adjust the transmission rate at each sensor to guarantee the packet loss ratio requirement. Jung et al. [29] proposed a novel contention-based MAC protocol in WBANs; the performance is evaluated by formulating the problem to be a discrete-time Markov chain model. Despite that the aforementioned studies made contributions to the development of WBANs, the lifetime of WBANs is still limited by the battery longevity. As an emergency solution to break the battery limitation in wireless networks, EH technology has attracted great attention from both industry and academia. In the work of Qiu et al. [30], a transmission power control scheme was proposed to improve the lifetime of the wind-powered wireless sensor networks (WSNs) by jointly considering the residual energy level and the amount of energy harvested. An optimal energy management strategy for solar-powered WSNs was proposed in the work of Niyato et al. [31]; the authors concentrated on the sleep and wakeup scheduling for energy conservation. However, different from EH-powered WSNs, EH-WBANs mainly harvest energy from human body bio-energy sources [32]. These human body bio-energy sources can be categorized into bio-chemical and bio-mechanical energy sources. The bio-chemical energy sources convert electrochemical to electricity for invasive body sensors, while bio-mechanical energy can be obtained from the locomotion of the human body [33]. In the work of Quwaider et al. [34], the weighted sum of the outage probabilities was the objective function to be minimized. The harvested energy was known as a priori to the scheduler, and an optimal offline algorithm was proposed to get the optimal solution. A joint power–QoS control scheme was proposed in the work of Ibarra et al. [35], namely powered by energy harvesting–quality of service (PEH–QoS). The PEH–QoS scheme combines three interconnected modules: the power–EH aware management module, the data queue aware control module, and the packet aggregator system. The core idea of PEH–QoS is to use the amount of power available for transmission and the amount of data stored in the queue to determine the maximum number of packets that can be transmitted in each data communication process. The simulation results show that the energy efficiency of the body sensor can be improved.

Looking at these previous works, the energy efficiency issue is rarely considered; even if some works tried to investigate EH-WBANs, they covered only limited aspects such as the sum-rate, transmission power, and the tradeoff between different objectives. In order to fill this gap, we investigate the energy efficient resource allocation scheme in EH-WBANs with the goal of maximizing the energy efficiency in this paper. This is also the motivation behind this work.

The main contributions of this paper are as follows:

- We consider a resource allocation problem for EH-WBANs with the goal of maximizing the average energy efficiency of body sensors. The resource allocation problem jointly considers the transmission mode, relay selection, allocated time slots, transmission power, and energy status to make the optimal allocation decision;
- We formulate the energy efficiency problem to be a discrete-time and finite-state Markov decision process (DFMDP) and a modified Q-learning algorithm, which reduces the state-action space in the original Q-learning algorithm, is proposed to solve the modeled problem;
- From the numerical analysis, we show that the proposed scheme can obtain the best energy efficiency and with the more rapid convergence speed by eliminating the irrelevant exploration space in the Q-table as compared with the classical Q-learning algorithm.

The remainder of this paper is organized as follows. In Section 2, the network model considered in this paper is presented. After that, the corresponding energy efficiency maximization problem is formulated and the proposed modified Q-learning algorithm is elaborated in Section 3. The simulation results are discussed in Section 4. Finally, we conclude the paper in Section 5.

## 2. Network Model Descriptions

In this section, we first depict the system model of the EH-WBAN, which is then followed by the details on the data transmission model, energy harvesting model, and energy efficiency model in EH-WBANs. Table 1 summarizes the different symbols and notations used throughout this paper.

### 2.1. Network Model

**Figure 1:**
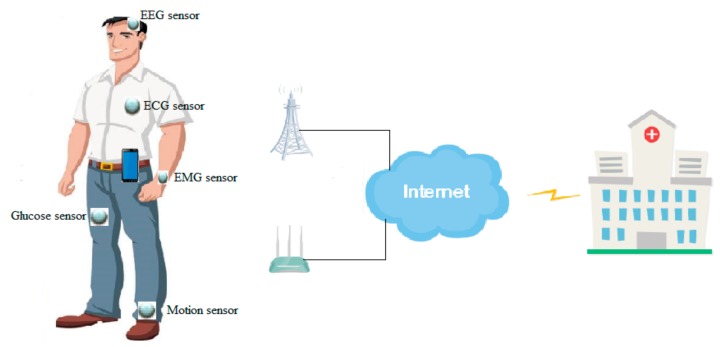
Scenario of the wireless body area network (WBAN). ECG: electrocardiogram sensor; EMG: electromyography sensor; EEG: electroencephalography sensor.

In this treatise, we consider a single EH-WBAN with one hub and multiple EH-powered body sensors, as illustrated in Figure 1. The hub is placed on the belt, while various body sensors, for example, the electrocardiogram sensor (ECG), the electromyography sensor (EMG), the electroencephalography sensor (EEG), the glucose sensor, and motion sensor, are placed in different positions of the body according to different detection purposes. For simplicity, we only consider the uplink transmission from body sensors to the hub and only body sensors are equipped with the EH function. Here, we denote the hub as *H* and body sensors as $S_{n},\;n\in \left(1,\;2,\;\ldots ,\;N\right)$. In this work, we assume that both direct transmission and cooperative transmission modes are supported by the network layer, as recommended by IEEE 802.15.6 standard [36]. In cooperative transmission mode, only two-hop transmission is permitted. We define a binary parameter $\alpha_{S_{n}}\in \left\{0,\;1\right\}\;n\in \left(1,\;2,\;\ldots ,\;N\right)$ to indicate the transmission mode that is utilized recently by the *n*-th body sensor. $\alpha_{S_{n}}=1$ denotes that the *n*-th body sensor transmits data to the hub directly, while $\alpha_{S_{n}}=0$ indicates that the *n*-th body sensor is in cooperative transmission mode. In the MAC layer, TDM technology is employed to prevent the mutual interference. As the slotted system model is adopted, each transmission frame can be divided into *K* number of time slots and the time slot set is denoted as $\psi =\left(1,\;2,\;\ldots ,\;K\right)$. We set $t_{0}=0$ and $t_{K}=T$. The duration of each slot is denoted as $\tau_{k}=t_{k}-t_{k-1}\;\forall k\in \psi$. In this model, these time slots can be assigned to the body sensors, whether they operate in direct transmission or cooperative transmission modes. Meanwhile, it should be noted that the transmission mode of each body sensor is determined by the resource allocation strategy in the proposed scheme. For example, after the proposed scheme finds the optimal resource allocation strategy, in which a specific body sensor is determined to transmit data in cooperative mode, then the body sensor will use the certain time slot (which is also determined by the resource allocation strategy) to transmit data in cooperative mode.

In case of direct transmission mode, we define a binary parameter $\beta_{S_{n}}^{k}\in \left\{0,\;1\right\},\;\left(n\in \left(1,\;2,\;\ldots ,\;N\right),\;\forall k\in \psi \right)$ to indicate which time slot is assigned to a specific body sensor. $\beta_{S_{n}}^{k}=1$ denotes that the *k*-th time slot is assigned to the *n*-th body sensor for direct transmission, while $\beta_{S_{n}}^{k}=0$ means the *k*-th time slot is not assigned to the *n*-th body sensor for direct transmission. More specifically, another two reasonable assumptions are made in this model: (1) the hub can only receive data from one body sensor at each time slot; (2) in each time frame, each body sensor only be assigned at most to one time slot for direct transmission. Thus, we can derive two constraints as Equations (1) and (2):

(1) $$\overset{N}{\underset{n=1}{\sum }}\beta_{S_{n}}^{k}\leq 1,\;k\in \psi ,$$

(2) $$\overset{K}{\underset{k=1}{\sum }}\beta_{S_{n}}^{k}\leq 1,\;n\in \left(1,\;2,\;\ldots ,\;N\right).$$

In the case of cooperative transmission mode, we assume that the *K* time slots in a time frame are allocated to both source-relay and relay-hub links. This assumption is mainly to be used to guarantee the fairness between direct transmission and cooperative transmission, to obtain the optimal resource allocation strategy. Similarly, we define a parameter $\delta_{S_{n}\to S_{m}}^{k}\in \left\{0,\;1\right\},\;\left(n,m\in \left(1,\;2,\;\ldots ,\;N\right),\;\forall k\in \psi \right)$ as an indicator that the *k*-th time slot is allocated to *n*-th body sensor for transmitting data to the *m*-th body sensor, which is selected as the relay node of the *n*-th body sensor. Meanwhile, $\delta_{S_{m}\to H}^{k}\in \left\{0,\;1\right\}\left(n,m\in \left(1,\;2,\;\ldots ,\;N\right),\;\forall k\in \psi \right)$ is denoted as the indicator that the *m*-th body sensor forwards the data from the *n*-th body sensor to the hub using the *k*-th time slot. In this mode, we assume that each source sensor can select one relay sensor and each relay sensor can only forward data from one source sensor at any time slot. Thus, we can obtain two constraints as Equations (3) and (4):

(3) $$\overset{N}{\underset{m=1,m\neq n}{\sum }}\delta_{S_{n}\to S_{m}}^{k}\leq 1,\;\;\;\;\overset{N}{\underset{n=1,n\neq m}{\sum }}\delta_{S_{n}\to S_{m}}^{k}\leq 1,$$

(4) $$\overset{N}{\underset{n=1,n\neq m}{\sum }}\delta_{S_{m}\to H}^{k}\leq 1,\;\;\;\;\;\overset{N}{\underset{m=1,m\neq n}{\sum }}\delta_{S_{m}\to H}^{k}\leq 1.$$

Furthermore, because each link can only be assigned at most to one time slot, we can obtain the constraint as Equation (5):

(5) $$\overset{K}{\underset{k=1}{\sum }}\delta_{S_{n}\to S_{m}}^{k},\;\overset{K}{\underset{k=1}{\sum }}\delta_{S_{m}\to H}^{k}\leq 1\;\;n\neq m.$$

Another aspect to note is that the data transmission from the source sensor to relay sensor should be prior to the transmission from the relay sensor to hub. Therefore, we can obtain Equation (6):

(6) $$\overset{x}{\underset{k=1}{\sum }}\delta_{S_{n}\to S_{m}}^{k}-\overset{K}{\underset{k=x+1}{\sum }}\delta_{S_{m}\to H}^{k}\geq 0,\;x\in \left(1,\;2,\;\ldots ,\;K-1\right).$$

### 2.2. Data Transmission Model

In WBANs, different body sensors with different monitoring purposes may have the heterogeneity of data rate requirement. In the proposed data transmission model, we suppose that all the body sensors can be served as the source node in direct transmission mode or relay node in cooperative mode. The selection of mode is determined by the resource allocation scheme. We denote $R_{n}$ as the data rate of the *n*-th body sensor, and it can be expressed as Equation (7):

(7) $$R_{n}=\alpha_{S_{n}}\cdot {R_{n}}^{d}+\left(1-\alpha_{S_{n}}\right)\cdot {R_{n}}^{c},\;n\in \left(1,\;2,\;\ldots ,\;N\right),$$

where ${R_{n}}^{d}$ is the data rate of the *n*-th body sensor in direct transmission mode, and ${R_{n}}^{c}$ denotes the data rate of the *n*-th body sensor in cooperative transmission mode. On the basis of the above analysis, we can derive the instantaneous signal to interference plus noise ratio (SINR) of direct transmission and cooperative transmission. Equations (8)–(10) give the instantaneous SINR of direct link, source-relay link, and relay-hub link in the *k*-th time slot, respectively.

(8) $$SINR_{n,k}^{d}=\frac{p_{n,k}^{d}\cdot g_{S_{n}\to H}}{\sum_{n_{1}=1,n_{1}\neq n}^{N}\sum_{m=1,\;m\;\neq \;n,n_{1}}^{N}\delta_{S_{n_{1}}\to S_{m}}^{k}\cdot p_{n_{1},m,k}^{s}\cdot g_{S_{n_{1}}\to S_{m}}+n_{0}},$$

where $p_{n,k}^{d}$ denotes the instantaneous transmission power of the *n*-th body sensor in the *k*-th time slot when transmitting data to the hub; $g_{S_{n}\to H}$ is the transmission gain between the *n*-th body sensor and the hub; $p_{n_{1},m,k}^{s}$ denotes the instantaneous transmission power of *n*_1_-th body sensor in the *k*-th time slot when transmitting data to *m*-th body sensor, which is selected as the relay sensor of the *n*_1_-th body sensor; $g_{S_{n_{1}}\to S_{m}}$ is the transmission gain between the *n*_1_-th body sensor and *m*-th body sensor; and $n_{0}$ is the noise power.

(9) $$SINR_{n,m,k}^{s\to r}=\frac{p_{n,m,k}^{s\to r}\cdot g_{S_{n}\to S_{m}}}{I_{n,m,k}^{s\to r}+n_{0}},$$

$$I_{n,m,k}^{s\to r}=\overset{N}{\underset{\begin{array}{c} n_{1}=1\\ n_{1}\neq n,m \end{array}}{\sum }}\overset{N}{\underset{\begin{array}{c} m_{1}=1\\ m_{1}\neq n,n_{1},m \end{array}}{\sum }}\delta_{S_{n_{1}}\to S_{m_{1}}}^{k}\cdot p_{n_{1},m_{1},k}^{s\to r}\cdot g_{S_{n_{1}}\to S_{m}},\;+\overset{N}{\underset{\begin{array}{c} n_{1}=1\\ n_{1}\neq n,m \end{array}}{\sum }}\beta_{S_{n_{1}}}^{k}\cdot p_{n_{1},k}^{d}\cdot g_{S_{n_{1}}\to S_{m},}+\overset{N}{\underset{\begin{array}{c} n_{1}=1\\ n_{1}\neq n,m \end{array}}{\sum }}\overset{N}{\underset{\begin{array}{c} m_{1}=1\\ m_{1}\neq n,n_{1},m \end{array}}{\sum }}\delta_{S_{m_{1}}\to H}^{k}\cdot p_{n_{1},m_{1},k}^{r\to H}\cdot g_{S_{m_{1}}\to S_{m}},$$

where $p_{n,m,k}^{s\to r}$ denotes the instantaneous transmission power of the *n*-th body sensor when transmitting data to the *m*-th body sensor, which is selected as its relay sensor in the *k*-th time slot; $p_{n,m,k}^{r\to H}$ denotes the instantaneous transmission power of *m*-th body sensor in the *k*-th time slot when forwarding data from *n*-th body sensor to the hub; and $I_{n,m,k}^{s\to r}$ is the total instantaneous interference of the source-relay link in the *k*-th time slot. The expression of $I_{n,m,k}^{s\to r}$ includes three items; the first item indicates the interference from other source-relay links, the second item is the interference from direct transmission between the source body sensor and hub, and the three item represents the interference from relay-hub links.

(10) $$SINR_{n,m,k}^{r\to H}=\frac{p_{n,m,k}^{r\to H}\cdot g_{S_{m}\to H},}{I_{n,m,k}^{r\to H}+n_{0}},$$

$$I_{n,m,k}^{r\to H}=\overset{N}{\underset{\begin{array}{c} n_{1}=1\\ n_{1}\neq m \end{array}}{\sum }}\overset{N}{\underset{\begin{array}{c} m_{1}=1\\ m_{1}\neq m,n_{1} \end{array}}{\sum }}\delta_{S_{n_{1}}\to S_{m_{1}}}^{k}\cdot p_{n_{1},m_{1},k}^{s\to r}\cdot g_{S_{n_{1}}\to S_{m}},$$

where $I_{n,m,k}^{r\to H}$ is the total instantaneous interference between the relay sensor and hub when the *m*-th body sensor is selected as the relay of the *n*-th body sensor in the *k*-th time slot.

Additionally, channel fading between body sensors and the hub is affected by many factors such as clothing and obstructions due to different body segments [37], thus the dynamic link characteristics should be taken into full consideration. In this paper, the channel fading takes into account both large-scale fading and small-scale fading. The channel gain g can be represented as Equation (11):

(11) g=α·h,

where α denotes the large-scale fading, which includes path loss and shadowing. It can be modeled as $\alpha =PL\cdot \beta {\left(d\right)}^{-\varphi }$. PL is the path loss constant; β denotes the log-normal shadowing random component; d is the distance between transmitter and receiver in a communication link; φ is the power decay exponent; and h is the small-scale fading, which is assumed as Rayleigh small-scale fading with unit mean.

According to Shannon’s theorem, we can obtain the transmission rate of direct mode as given in Equation (12):

(12) $${R_{n}}^{d}=\overset{K}{\underset{k=1}{\sum }}\beta_{S_{n}}^{k}\cdot B\cdot log_{2}\left(1+SINR_{n,k}^{d}\right),$$

when B denotes the bandwidth of the channel.

The transmission rate of the cooperative mode ${R_{n}}^{c}$ can be divided into two parts: one is the transmission rate of source-relay link ${R_{n}}^{c,\;s\to r}$ and another is the transmission rate of relay-hub link ${R_{n}}^{c,\;r\to H}$, as shown in Equations (13) and (14):

(13) $${R_{n}}^{c,\;\;s\to r}=\overset{N}{\underset{\begin{array}{c} m=1\\ m\neq n \end{array}}{\sum }}\overset{K}{\underset{k=1}{\sum }}\delta_{S_{n}\to S_{m}}^{k}\cdot B\cdot log_{2}\left(1+SINR_{n,m,k}^{s\to r}\right),$$

(14) $${R_{n}}^{c,\;r\to H}=\overset{N}{\underset{\begin{array}{c} m=1\\ m\neq n \end{array}}{\sum }}\overset{K}{\underset{k=1}{\sum }}\delta_{S_{m}\to H}^{k}\cdot B\cdot log_{2}\left(1+SINR_{n,m,k}^{r\to H}\right).$$

However, in cooperative transmission mode, the transmission rate of the path between the source body sensor and hub is limited by the smaller transmission rate of the source-relay link and relay-hub link. Hence, the transmission rate of the cooperative mode is ${R_{n}}^{c}=min\;\left({R_{n}}^{c,\;s\to r},\;{R_{n}}^{c,\;r\to H}\right)$.

### 2.3. Data Serving Model

In this scenario, we make the assumption that the data are stored in the form of packets in the buffer of the device. The arrived data at each body sensor follow an independently and identically distributed (i.i.d.) sequence with an average rate of $\lambda_{d}$ [38]. Practically, we assume that the buffer of the device is finite and served in first in first out fashion. We denoted $DQ_{S_{n}}^{k}$ as the instantaneous data queue length at the *n*-th body sensor in time slot *k*. The maximum traffic queue length of body sensors is represented by $\;DQ_{S_{n}}^{max}$. Accordingly, we can obtain the update function of the instantaneous data queue length as Equation (15):

(15) $$DQ_{S_{n}}^{k}=\;min\left\{\;DQ_{S_{n}}^{max},\;DQ_{S_{n}}^{k-1}-min\left\{\lfloor \frac{\alpha_{S_{n}}\cdot R_{n}{}^{d}+\left(1-\alpha_{S_{n}}\right)\cdot {R_{n}}^{c}}{PS_{data}}\rfloor ,\;DQ_{S_{n}}^{k-1}\right\}+A_{S_{n}}^{k-1}\right\}$$

where $PS_{data}$ is the traffic packet size, $\frac{\alpha_{S_{n}}\cdot R_{n}{}^{d}+\left(1-\alpha_{S_{n}}\right)\cdot {R_{n}}^{c}}{PS_{traffic}}$ is the instantaneous service rate of transmission link of *n*-th body sensor in *k* − 1-th time slot, and $A_{S_{n}}^{k-1}$ is the arriving traffic packets of the *n*-th body sensor in the *k* − 1-th time slot.

### 2.4. Energy Harvesting Model

We denoted $E_{n,\;k}$ as the energy harvested by the *n*-th body sensor in the *k*-th time slot. $\{E_{n,\;1},{\;E}_{n,\;2},\;\ldots ,{\;E}_{n,\;t},\;\ldots ,\;E_{n,\;K}$} is the time sequence of energy harvested in a transmission frame. It is also i.i.d. sequence with average rate of $\lambda_{e}$ [38]. We denote $EQ_{S_{n}}^{k}$ as the instantaneous energy queue length of the *n*-th body sensor in the *k*-th time slot. The maximum energy queue length of body sensors is represented by $EQ_{S_{n}}^{max}$. Therefore, we can obtain the update function of the instantaneous energy queue length as Equation (16):

(16) $$EQ_{S_{n}}^{k}=min\left\{EQ_{S_{n}}^{max},\;Q_{S_{n}}^{k-1}-min\left\{\left\lceil \frac{p_{n,k-1}}{PS_{energy}}\right\rceil ,\;EQ_{S_{n}}^{k-1}\right\}+E_{n,\;k-1}\right\},$$

where $PS_{energy}$ is the energy packet size with the unit of Joules/packet. $p_{n,k-1}$ denotes the transmission power of the body sensor in the *k* − 1-th time slot. According on the transmission mode, $p_{n,k-1}$ can be set to one of $\;p_{n,k-1}^{d}$, $p_{n,m,k-1}^{s\to r}$, and $p_{n,m,k-1}^{r\to H}$.

It is worth noting that, because the capacity of the energy storage device is finite, two constraints can be derived from Equation (15), as expressed in Equations (17) and (18):

(17) $$\overset{K}{\underset{k=1}{\sum }}\left\lceil \frac{p_{n,k-1}}{PS_{energy}}\right\rceil \leq \overset{K}{\underset{k=1}{\sum }}EQ_{S_{n}}^{k},\;\forall K\in \left\{1,2,\ldots \right\},$$

(18) $$\overset{K}{\underset{k=1}{\sum }}EQ_{S_{n}}^{k}-\overset{K}{\underset{k=1}{\sum }}\left\lceil \frac{p_{n,k-1}}{PS_{energy}}\right\rceil \leq EQ_{S_{n}}^{max},\;\forall K\in \left\{1,2,\ldots \right\}.$$

Equation (16) depicts that the current available energy cannot exceed the total energy in the battery. Equation (17) expresses that the total energy stored in the battery cannot exceed the maximum battery capacity.

### 2.5. Energy Efficiency Model

In this paper, we define the energy efficiency (EE) of WBANs as the ratio of the transmission rate to the consumed transmission power. Equation (19) gives the energy efficiency of the *n*-th body sensor in the *k*-th time slot.

(19) $$EE_{S_{n}}^{k}=\frac{\alpha_{S_{n}}\cdot {R_{n}}^{d}+\left(1-\alpha_{S_{n}}\right)\cdot {R_{n}}^{c}}{p_{n,k}}\;\;\forall n\in \left(1,\;2,\;\ldots ,\;N\right),\;\forall k\in \psi$$

Therefore, the average energy efficiency of the overall WBANs is presented as follows:

(20) $$EE=\frac{1}{N}\cdot \overset{K}{\underset{k=1}{\sum }}\overset{N}{\underset{N=1}{\sum }}\;\;EE_{S_{n}}^{k}.$$

The corresponding EE optimization problem can be formulated as follows:

(21) $$\begin{array}{c} maximize\\ \alpha_{S_{n}},\;\beta_{S_{n}}^{k},\delta_{S_{n}}^{k},\;p_{n,k},\;\;\; \end{array}\;\;\;\;\;\;\;\;EE,$$

subject to:

$$\overset{N}{\underset{n=1}{\sum }}\beta_{S_{n}}^{k}\leq 1,\;k\in \;\psi ,\;\overset{K}{\underset{k=1}{\sum }}\beta_{S_{n}}^{k}\leq 1,\;n\in \left(1,\;2,\;\ldots ,\;N\right),\;\overset{N}{\underset{m=1,m\;\neq \;n}{\sum }}\delta_{S_{n}\to S_{m}}^{k}\leq 1,\;\overset{N}{\underset{n=1,n\neq m}{\sum }}\delta_{S_{n}\to S_{m}}^{k}\leq 1,\;\overset{N}{\underset{n=1,n\neq m}{\sum }}\delta_{S_{m}\to H}^{k}\leq 1,\;\overset{N}{\underset{m=1,m\neq n}{\sum }}\delta_{S_{m}\to H}^{k}\leq 1,\;\overset{K}{\underset{k=1}{\sum }}\delta_{S_{n}\to S_{m}}^{k},\;\overset{K}{\underset{k=1}{\sum }}\delta_{S_{m}\to H}^{k}\leq 1\;n\neq m,\;\overset{x}{\underset{k=1}{\sum }}\delta_{S_{n}\to S_{m}}^{k}-\overset{K}{\underset{k=x+1}{\sum }}\delta_{S_{m}\to H}^{k}\geq 0,\;\forall x\in \left(1,\;2,\;\ldots ,\;K-1\right),\;\overset{K}{\underset{k=1}{\sum }}\left\lceil \frac{p_{n,k-1}}{PS_{energy}}\right\rceil \leq \overset{K}{\underset{k=1}{\sum }}EQ_{S_{n}}^{k},\;\forall K\in \left\{1,2,\ldots \right\},\;\overset{K}{\underset{k=1}{\sum }}EQ_{S_{n}}^{k}-\overset{K}{\underset{k=1}{\sum }}\left\lceil \frac{p_{n,k-1}}{PS_{energy}}\right\rceil \leq \;EQ_{S_{n}}^{max},\;\forall K\in \left\{1,2,\ldots \right\},\;p_{n,k}^{d}\leq p_{n}^{max}\;\forall n\in \left(1,\;2,\;\ldots ,\;N\right),\;\forall k\in \psi ,\;p_{n,m,k}^{s\to r}\leq p_{n}^{max}\;n,m\in \left(1,\;2,\;\ldots ,\;N\right),\;n\neq m,\forall k\in \psi ,\;p_{n,m,k}^{r\to H}\leq p_{n}^{max}n,m\in \left(1,\;2,\;\ldots ,\;N\right),\;n\neq m,\forall k\in \psi .$$

## 3. Problem Formulation and Optimization Algorithm

From the energy efficiency maximization problem, we can see that it is a long-term multi-objective optimization problem. Simultaneously, because the variables $p_{n,k}$ are continuous, while $\alpha_{S_{n}},\;\beta_{S_{n}}^{k},\;\mathrm{and}\;\delta_{S_{n}}^{k}$ are binary, problem (21) is a mixed integer nonlinear programming problem, which cannot be directly solved by convex optimization methods. Even if we can transform the original problem into a tractable convex optimization problem, the problem still requires the prior network information such as channel state information (CSI) to achieve optimal performance. However, WBANs normally work in a dynamic channel characteristic owing to the posture and environment variation [39,40]. Furthermore, from Equation (15), we found that the current consumed energy packets are only related to current arrivals and the previous remainders in the energy queue. Thus, we can formulate problem (21) as the discrete-time and finite-state Markov decision process (DFMDP) [41]. More specifically, in this work, we formulate our scenario into a centralized DFMDP. Therefore, the hub should acquire all information about both the network and users to make the optimal decision. The reasons for formulating the centralized DFMDP are as follows: (1) the hub has more abundant resources compared with the body sensor; (2) the centralized DFMDP will reduce the network signaling overhead and redundancy as compared with distributed DFMDP. In distributed DFMDP, each body sensor should make the decision without the complete knowledge and global network information that will increase the computation complexity and consume more energy. Meanwhile, owing to the high computation complexity, we propose to utilize a modified Q-learning algorithm to solve the optimization problem.

### 3.1. DFMDP Model

DFMDP is a discrete time stochastic control process that provides a mathematical framework for modeling decision-making problems in uncertain and stochastic environments [42]. Typically, a DFMDP is defined by a tuple (*S, A, p, r*), where *S* is a finite set of states, *A* is a finite set of actions, *p* is a transition probability from state *s* to state *s’* (∀s∈S,∀s’∈S) after action *a* $\left(\forall a\in A\right)$ is performed, and *r* is the immediate reward obtained after *a*$\;\left(\forall a\in A\right)$ is executed. We denote π as a policy that is a mapping from a state to an action. Our goal is to find the optimal policy denoted as $\pi^{*}$ to maximize the reward function over a finite time horizon in the DFMDP. Therefore, the detailed tuple in our proposed model is designed as follows:

1. The state of each body sensor $S_{n}$ in the *k*-th time slot can be denoted as $Sta_{S_{n}}^{k}\in S$. In this model, $Sta_{S_{n}}^{k}$ contains two parts: $DQ_{S_{n}}^{k}$ and $EQ_{S_{n}}^{k}$. They are the data and energy queue lengths of the *n*-th body sensor at the beginning of the *k*-th time slot, respectively. To ensure the completeness of the exploration of state space, $DQ_{S_{n}}^{k}$ and $EQ_{S_{n}}^{k}$ are specified to be an integer and take the values of $\left[0,\;1,\;\ldots ,\;DQ_{S_{n}}^{max}\right]$ and $\left[0,\;1,\;\ldots ,\;EQ_{S_{n}}^{max}\right]$, respectively.
2. The action *a*$\;\left(\forall a\in A\right)$ in this scenario should be the resource allocation variables, which include transmission mode $\alpha_{S_{n}}$, time slot allocation $\beta_{S_{n}}^{k}$, relay selection $\delta_{S_{n}}^{k}$, and power allocation $p_{n,k}$. To make sure the integrity of the exploration of action space, $p_{n,k}^{d}$, $p_{n,m,k}^{s\to r}$, and $p_{n,m,k}^{r\to H}$ should be subject to the maximum transmission power $p_{n}^{max}$.
3. Obviously, the reward *r* is the immediate reward corresponding to current state–action pair, which is given by Equation (20).

However, the traditional value-based algorithms such as Monte Carlo [43] and temporal difference (TD) [44] algorithms have some shortcomings in practical applications, for instance, they cannot handle the tasks in continuous action space efficiently and the final solution may not be globally optimal. Therefore, we intend to adopt a policy-based algorithm in this paper.

In order to address the formulated DFMDP problem, the Q-learning algorithm is an effective tool [45]. The core idea behind the Q-learning algorithm is to first define the value function $V^{\pi }\left(s_{d_{j}}^{k}\right)\to r$ that represents the expected value gotten by policy π from each state $s_{d_{j}}^{k}\in S$. The value function $V^{\pi }$ for policy π quantifies the goodness of the policy via an infinite horizon and discounted MDP, which can be represented as Equation (22):

(22) $$\begin{array}{cc} V^{\pi }\left(s\right)=E_{\pi } & \;[\overset{\infty }{\underset{k=0}{\sum }}\gamma \cdot r^{k}\left(s^{k},a^{k}\right)|s^{0}=s]\\ & =E_{\pi }\left[r^{k}\left(s^{k},a^{k}\right)+\gamma \cdot V^{\pi }\left(s^{k+1}\right)|s^{0}=s\right]. \end{array}$$

Additionally, it should be noted that, to solve the MDP problem with discrete state and action spaces, the Q-learning algorithm is capable of obtaining the optimal policy [42]. Because we aim to find the optimal policy $\pi^{*}$, the optimal action at each state can be found by means of the optimal value function, as in Equation (23):

(23) $$V^{*}\left(s\right)=\begin{array}{c} max\\ a^{k} \end{array}\left\{E_{\pi }\left[r^{k}\left(s^{k},a^{k}\right)+\gamma \cdot V^{\pi }\left(s^{k+1}\right)\right]\right\}.$$

If we denoted $Q^{*}\left(s,a\right)≜r^{k}\left(s^{k},a^{k}\right)+\gamma \cdot E_{\pi }\left[V^{\pi }\left(s^{k+1}\right)\right]$ as the optimal Q-function for all state–action pairs, the optimal value function can be rewritten by $V^{*}\left(s\right)=\begin{array}{c} max\\ a \end{array}\left\{Q^{*}\left(s,a\right)\right\}$. The $Q^{*}\left(s,a\right)$ can be obtain through the iterative process according to the Equation (24):

(24) $$Q^{k+1}\left(s^{k},a^{k}\right)=Q^{k}+\alpha \left[r^{k}\left(s^{k},a^{k}\right)+\gamma maxQ^{k}\left(s^{k},a^{k+1}\right)-Q^{k}\left(s^{k},a^{k}\right)\right],$$

where α is the learning rate to determine the impact of new information to the existing Q-value, and $\gamma \in \left[0,1\right]$ is the discount factor. Algorithm 1 gives the pseudo-code of the Q-learning algorithm.
**Algorithm 1** The Q-learning based resource allocation algorithminitialize the table entry $Q\left(s,a\right)$ arbitrarily for each state-action pair $\left(s,a\right)$
observe the current state s, initialize the value of α and γ
**for***episode = 1 to M***do**  from the current state-action pair $\left(s,a\right)$, execute action a and obtain  the immediate reward r and a new state $s^{\prime }$
  select an action $a^{\prime }$ based on the state $s^{\prime }$ and update the table entry for  $Q\left(s,a\right)$ as expressed in Equation (18)  replace $s\leftarrow s^{\prime }$**end for****Output:**$\pi^{*}\left(s\right)=argmax_{a}Q^{*}\left(s,a\right)$

Initially, the resource allocation scheme randomly selects a transmission mode, relay node (if necessary), time slot, and transmission power without the consideration of the data and energy queue status in each body sensor to get an initial state–action pair. Meanwhile, the algorithm initializes the value of α and γ. After the first iteration, from current state–action pair, we can obtain an immediate reward and a new state (the data and energy queue lengths of each body sensor). Then, the algorithm updates the state–action pair, as expressed by Equation (19). Once either all Q-values or a certain number of iterations is reached, the algorithm will terminate. The optimal policy indicating an action (resource allocation scheme) to be taken at each state is maximized for all states. However, the convergence speed of the classical Q-learning algorithm may not be able to find the optimal policy within the acceptable time, especially in the practical and complicated model. Thus, one aspect that needs to be further considered is the necessity of cut down of the original state–action space of the Q-learning algorithm, as the convergence speed is sensitive to the size of the state–action space [46]. Therefore, we proposed a modified Q-learning algorithm, which aims to improve the convergence speed by cutting down the irrelevant state–action pairs.

### 3.2. The Proposed Modified Q-Learning Algorithm

In order to ensure the integrity of the exploration space, the values of state and action are set to an integer from 0 to the maximum value. However, this assumption will result in unnecessary exploring of state-action pairs in the original space. The proposed modified Q-learning algorithm intends to cut down these irrelevant pairs from both the irrelevant state and irrelevant action. In the irrelevant state aspect, we define the valid state space that should be explored to simultaneously achieve two requirements: available energy and serviceable data. Table 2 demonstrates the valid state space that should be explored. In Table 2, * represents the valid state and 0 indicates the invalid state. Nevertheless, in the view of the valid state, all the actions should be explored. Moreover, we investigate the irrelevant action in this work.

Similarly, in the irrelevant action aspect, according to exploration space integrity, the value of one action in the Q-table is as follows: $p_{n,k}$ ranges from 0 to $p_{n}^{max}$. However, the value of $p_{n}^{max}$ may be larger than the current available energy $p_{n,k}$. In this case, the range of action value can be cut down from 0 to $p_{n,k}$. In this way, we can further reduce the state–action space.

To better illustrate the computation complexity, we compare the state–action space size of the proposed modified Q-learning based algorithm with the classical Q-learning algorithm. We assume that the maximum available energy and serviceable data in each sensor is *x* packets and the maximum power control parameter in each sensor is *y* packets. On the basis of the definition of the state–action place, Table 3 gives the computation complexity of the proposed modified Q-learning based algorithm and classical Q-learning algorithm.

From Table 3, we can see that the computation complexity of the classical Q-learning algorithm increases exponentially with the number of sensors. However, the state–action space size of the proposed modified Q-learning based algorithm is completely free from the influence of sensor numbers.

Meanwhile, the proposed modified Q-learning scheme also contributes to the balance of energy consumption among body sensors. This is because the proposed modified Q-learning scheme considered the amount of harvested energy of each body sensor while allocating resources. In such a situation, if a specific body sensor harvests less energy from the environment, it will not be selected as relay. Moreover, the standard deviation of the consumed energy is less, which indicates that the consumed energy is distributed among body sensors and the lifetime of the overall WBANs can be extended.

## 4. Simulation Results and Analysis

In this section, we compare the proposed algorithm with other three schemes: (1) a random power allocation scheme; (2) the classical Q-learning resource allocation scheme; and (3) a joint power–QoS control scheme proposed in the literature [35]. To verify the effectiveness of the proposed algorithm, we evaluate the performance in terms of energy efficiency and convergence speed.

### 4.1. Simulation Setting

In simulations, we consider a WBAN scenario in which multiple heterogeneous body sensors and one hub are deployed with different positions for various detection purposes. Five typical body sensors with their initial energy are considered. They are as follows: ECG with initial energy of 20 mJ, EMG with initial energy of 12 mJ, EEG with initial energy of 16 mJ, glucose sensor with initial energy of 12 mJ, and motion sensor with initial energy of 18 mJ. For simplicity, we assume that the current energy harvesting technology is able to provide the required conversion efficiency. The hub is placed at the center of this topology with a communication range of 10 m, and it knows all the position information of the body sensors. Each body sensor is randomly placed in the topology with the communication range of 2–5 m [47]. Simultaneously, we suppose that only body sensors are equipped with the energy harvesting function, and the energy harvesting process is Poisson-distributed with a rate $\lambda_{e}$ at arrival instants $t_{k}$. The data arriving process is also Poisson-distributed with a rate $\lambda_{d}$ at arrival instants $t_{k}$. Moreover, the proposed modified Q-learning algorithm has no prior knowledge about them. Meanwhile, because the scenario contains lots of instability, we set 200 time instants for one episode, and the energy efficiency will be averaged to reduce the instability. For each configuration, we generate 100 independent runs and average the performance of energy efficiency. All of the detailed simulation variables used in this paper are summarized in Table 4.

### 4.2. Results and Analysis

The influence of learning rate α and discount factor γ on energy efficiency

**Figure 2:**
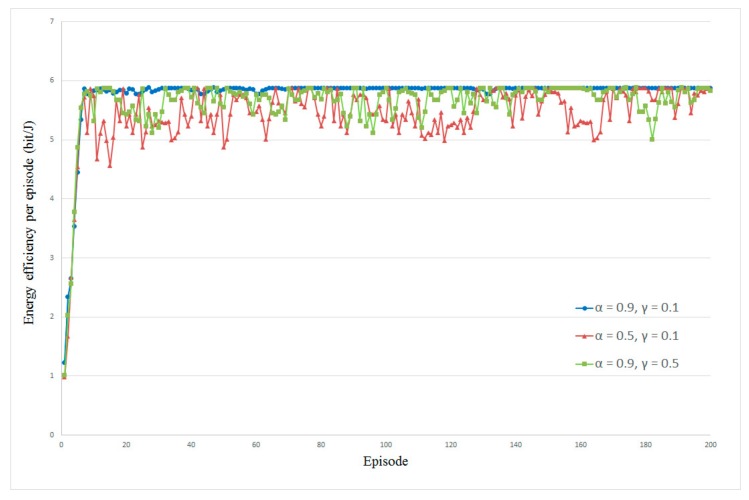
Influence of α and γ on energy efficiency in direct transmission mode.

**Figure 3:**
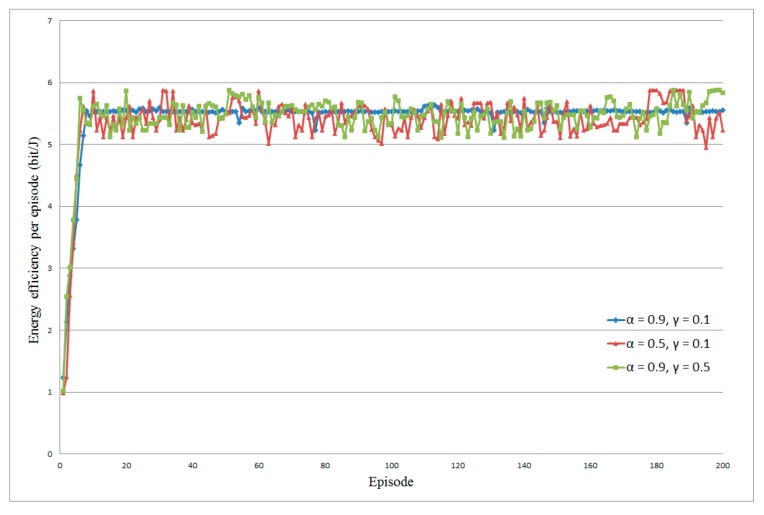
Influence of α and γ on energy efficiency in cooperative transmission mode.

In order to avoid other factors influencing the performance, we first evaluate the influence of learning rate α and discount factor γ on energy efficiency. We implement two scenarios in which one body sensor in direct transmission mode and two body sensors in cooperative transmission mode are deployed, respectively. The energy harvesting rate $\lambda_{e}$ is set to 3 packet/s and the data arriving rate $\lambda_{t}$ is set to 5 packet/s. Figure 2 and Figure 3 show the average energy efficiency under different values of α and γ. From the results of both scenarios, we can see that either the decrease of learning rate α or the increase of discount factor γ will cause the instability of energy efficiency in the proposed resource allocation algorithm. These two cases are depicted as brown and green marks in Figure 2 and Figure 3. This is because a smaller α leads to less exploration; in this case, the proposed algorithm increasingly concentrates on the greedy action, which has a more immediate effect in increasing the users’ utility. Contrarily, a larger γ causes less foresight in the policy updating, which will reduce the average utility in the long term [48]. From the blue marks in Figure 2 and Figure 3, we can observe that, while the α is set to 0.9 and γ is set to 0.1, the average energy efficiency is more stable, which means that the algorithm has the highest convergence speed. Furthermore, we also tried some more complex scenarios in which more sensors are deployed, but the influences of learning rate α and discount factor γ are similar. For simplicity and ease of understanding, we only demonstrate this scenario and we can obtain a vivid result that the proposed algorithm performs better in the case of a higher α and lower γ. Consequently, we set α=0.9 and γ=0.1, respectively, in the following simulations.

#### 4.2.1. Comparison between the Proposed Algorithm and Classical Q-Learning Algorithm

**Figure 4:**
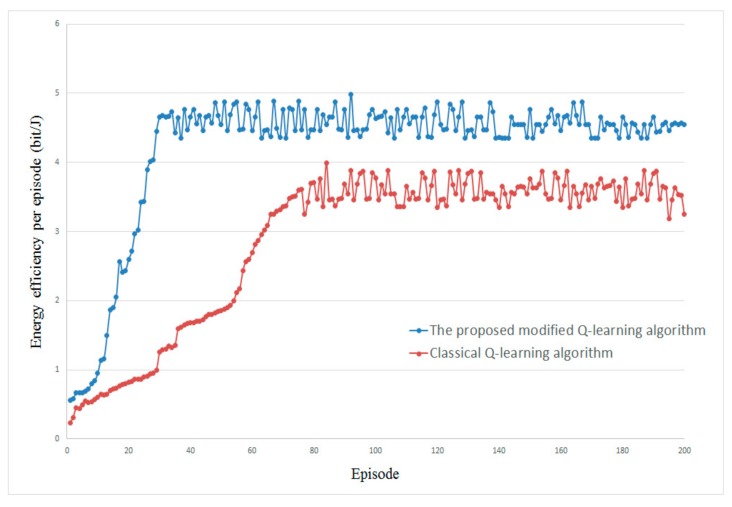
The optimization process for energy efficiency.

Figure 4 illustrates the optimization processes for energy efficiency of the proposed modified Q-learning algorithm and classical Q-learning algorithm. The simulation result gives two observations. First, after 30 episodes, the proposed modified algorithm trends to convergence rather than 80 episodes of classical Q-learning algorithm. This is because the proposed modified algorithm eliminates the irrelevant state and action spaces that reduce the exploration space. Hence, the convergence speed is accelerated. Second, as the episodes increase, the performance of the classical Q-learning algorithm trends to stable. However, the proposed algorithm outperforms the classical Q-learning algorithm over approximately 20%. This is because of the lower computation complexity and signaling overhead in the proposed algorithm.

#### 4.2.2. The Influence of the Number of Body Sensors Deployed

**Figure 5:**
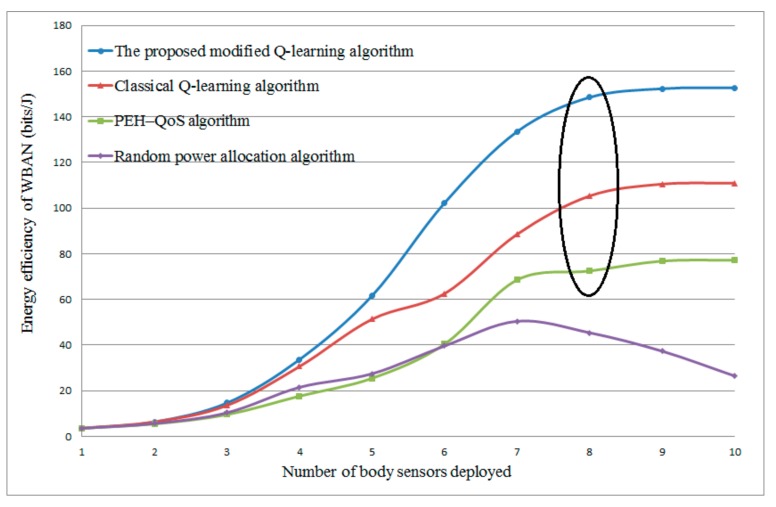
Energy efficiency versus different numbers of body sensor. PEH: powered by energy harvesting; QoS: quality of service.

Figure 5 presents the average energy efficiency for a different number of body sensors deployed in WBAN. From the result, it can be observed that the proposed algorithm has the highest energy efficiency among the classical Q-learning algorithm, the PEH–QoS algorithm proposed in the literature [35], and the random power allocation algorithm. For the Q-learning based algorithm and the PEH–QoS algorithm, as the number increases, the average energy efficiency is increased. This is because of the fact that more body sensors will lead to a higher data rate. However, it also can be observed that, as the number increases to 9, the average energy efficiency tends to stable. This is because these three algorithms all take available energy into consideration when allocating transmission power. For the random power allocation algorithm, the energy efficiency goes up when the number of body sensors is less than 7. As more body sensors are deployed, the energy efficiency is reduced, while it reaches a saturation point for most algorithms (except the random power allocation algorithm). This is because more body sensors deployed will involve more mutual interference, which further increases the transmission power.

#### 4.2.3. The Standard Deviation of Consumed Energy of Each Body Sensor

**Figure 6:**
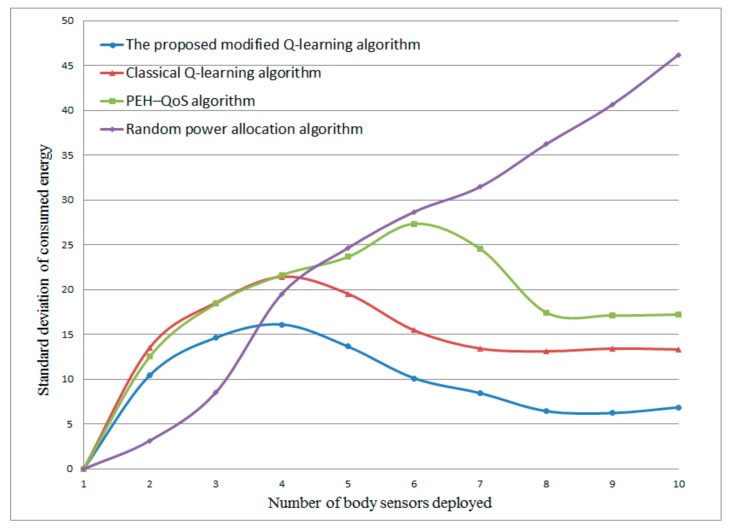
Standard deviation of consumed energy versus different numbers of body sensors.

Figure 6 presents the standard deviation of consumed energy versus the different number of body sensors deployed. The proposed modified Q-learning scheme gives the best performance with an average standard deviation of 10.33, compared with 15.65 using the classical Q-learning scheme, 19.98 using the PEH–QoS scheme from the literature [35] and 26.57 using the random power allocation scheme. This is because of the fact that the proposed modified Q-learning scheme is able to maintain the fairness of each body sensor by means of selecting the optimal transmission mode and relay body sensors to distribute the energy consumption to the relay sensors with more residual energy. It can also be observed that, at the beginning, there is an increase in the standard deviation as the number of body sensors increases. This is because, at the initial time, the number of body sensors is small and the consumed energy in each body sensor is unbalanced. After a further increase in the number of body sensors, the standard deviation tends to decrease and becomes stable. Another interesting finding is that the standard deviation of energy consumed of the random power allocation scheme increased sharply with the number of body sensors. This is because this scheme allocates transmission power randomly and does not consider the available energy residual in each body sensor.

#### 4.2.4. The Influence of Energy Harvesting Rate $\lambda_{e}$ and Data Arrival Rate $\lambda_{d}$

**Figure 7:**
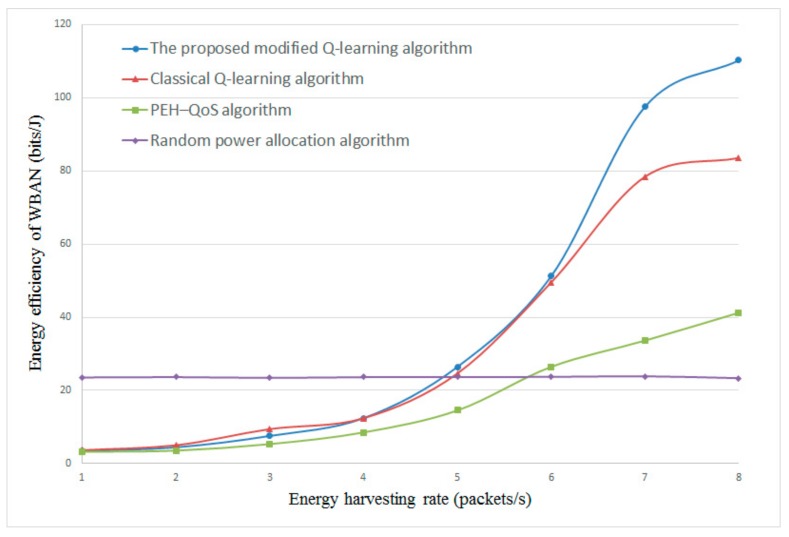
Energy efficiency versus energy harvesting rate $\lambda_{e}$ with constant $\lambda_{d}=5.$

**Figure 8:**
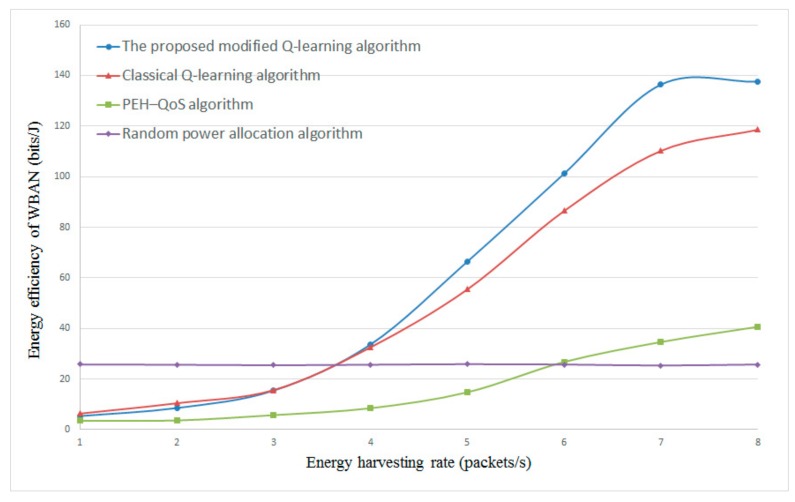
Energy efficiency versus energy harvesting rate $\lambda_{e}$ with constant $\lambda_{d}=8.$

Figure 7 and Figure 8 present the energy efficiency with different energy harvesting rates $\lambda_{e}$. The data arrival rate $\lambda_{d}$ is set to 5 and 8, respectively. From the results in Figure 7 and Figure 8, it is clear that the proposed modified Q-learning algorithm and classical Q-learning algorithm can achieve higher energy efficiency as compared with the PEH–QoS scheme from the literature [35] and the random power allocation scheme. With the increase of $\lambda_{e}$, the energy efficiency is improved sharply. This is because more energy can be harvested in each time slot with the higher $\lambda_{e}$, and the Q-learning-based algorithms are able to obtain an optimal correlation between energy harvesting time, transmission mode, relay selection, and power allocation. The PEH–QoS scheme proposed in the literature [35] gives a slightly better performance than the random power allocation scheme. However, another interesting finding is that, when the $\lambda_{e}$ is less than 4, the random power allocation scheme has the best energy efficiency. This is because the random power allocation scheme does not take into account the available energy when allocating transmission power.

**Figure 9:**
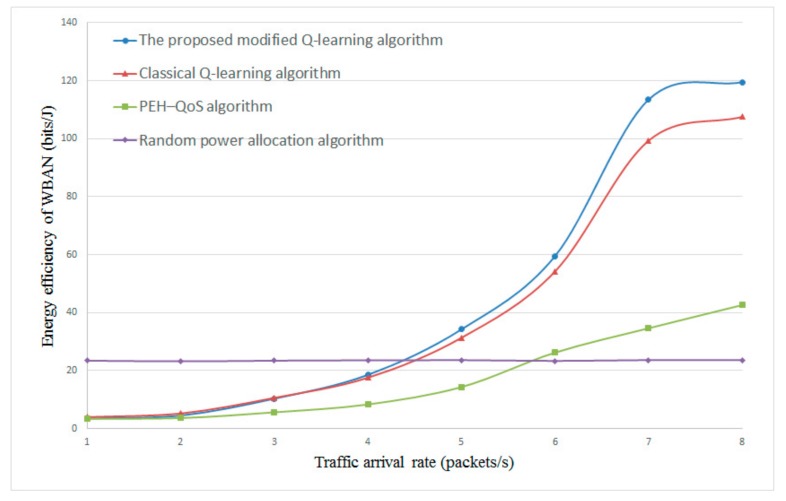
Energy efficiency versus traffic arrival rate $\lambda_{t}$ with constant $\lambda_{d}=3.$

**Figure 10:**
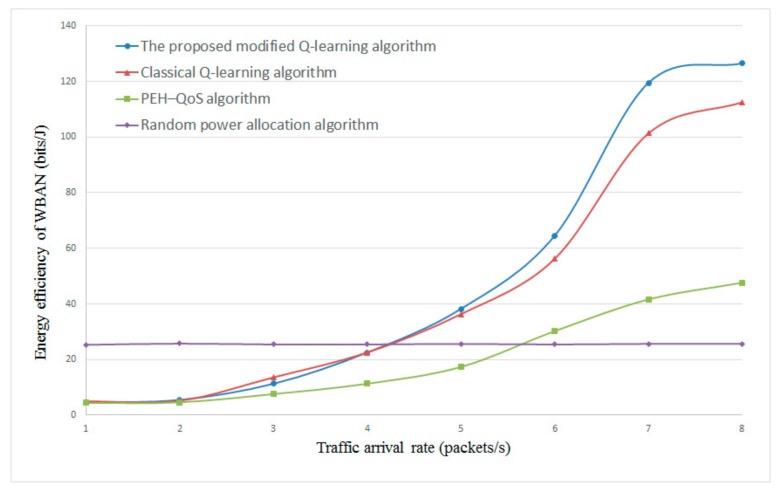
Energy efficiency versus traffic arrival rate $\lambda_{t}$ with constant $\lambda_{d}=5.$

Figure 9 and Figure 10 plot the energy efficiency with the different data arrival rate $\lambda_{d}$. In this scenario, the energy harvesting rate $\lambda_{e}$ is set to 3 and 5, respectively. As shown in Figure 9 and Figure 10, the proposed algorithm still obtains the highest energy efficiency in the comparison of the four algorithms along with the $\lambda_{d}$. The reason is that more data arrival will increase the transmission rate; in the meantime, the proposed algorithm can acheive the optimal coupling relationship between the transmission rate and energy consumption, thus improving the performance of energy efficiency. From the results of Figure 9 and Figure 10, we also observe that, as $\lambda_{d}$ achieves 7, the energy efficiency trends to stable; this is because more data to be served requires more transmission power $p_{n,k}$, but the $\lambda_{e}$ is constantly set to 3 and 5 in this simulation. Hence, the energy efficiency is going to stable. However, although the PEH–QoS algorithm from the literature [35] considered energy harvesting in resource allocation strategy, and the authors have also shown the performance with different transmission rates up to 1 Mbps, it did not take into consideration the data arrival rate of each body sensor. Thus, the results of the PEH–QoS algorithm in Figure 9 and Figure 10 are similar. Meanwhile, because the random power allocation scheme allocates transmission power randomly, the energy efficiency does not change with $\lambda_{e}$ and $\lambda_{d}$.

**Figure 11:**
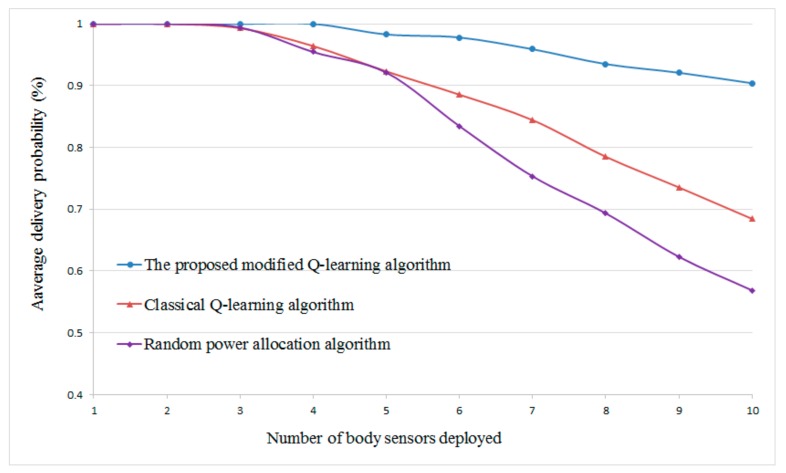
Average delivery probability of WBANs versus different numbers of body sensor.

In addition, because the WBANs concentrate mainly on the stringent reliability requirement for the safety-critical information. In this simulation, we also evaluate the reliability of the proposed scheme. The reliability is represented by the delivery probability. Specifically, we define the delivery probability as the probability of successfully delivering the sensory data of each body sensor with the size of *P* bits within an acceptable time *T*. Hence, the delivery probability can be given as $Prb\left\{\alpha_{S_{n}}\cdot R_{n}{}^{d}+\left(1-\alpha_{S_{n}}\right)\cdot R_{n}{}^{c}\geq \frac{P}{T}\right\}$. Figure 11 gives the average delivery probability of the WBANs, while different numbers of body sensors are deployed. We set *P* for each body sensor with the constant size of 1 Mb. From the result, we can find that, as the number of body sensors increases, the average delivery probabilities decrease for all schemes. This is because more body sensors being deployed will cause more mutual interference. However, the proposed scheme still gives better performance compared with the other two schemes throughout the tested cases. Remarkably, the proposed scheme is capable of guaranteeing the average delivery probability above 90% even in the worst case. Moreover, in conjunction with the result from Figure 5, we can validate the practicability of the proposed scheme.

## 5. Conclusions

The main motivation of this paper is to study the resource allocation scheme for EH-WBANs. Unlike the traditional WBANs, the available energy will be another vital issue that should be considered in the resource allocation scheme. Specifically, with the goal of maximizing the average energy efficiency, we formulate the resource allocation problem to be a DFMDP, in which the transmission mode, relay selection, allocated time slot, power allocation, and energy constraint of each body sensor are considered. Owing to the high complexity of the problem, we solve the maximization problem using a modified Q-learning algorithm. Through extensive simulations, it is shown that the proposed scheme enhances the energy efficiency significantly for different network settings. Additionally, with the conjunction of transmission reliability, we validate the practicability of the proposed scheme in EH-WBANs.

## Figures and Tables

**Table 1:** Table of notations. SINR: signal to interference plus noise ratio.

Symbol	Definition
*H*	Hub
$S_{n}$	*n*-th body sensor $n\in \left\{1,\;2,\;\ldots ,\;N\right\}$
ψ	Time slot $\psi =\left(1,\;2,\;\ldots ,\;K\right)$
$\tau_{k}$	*k*-th time slot
$\alpha_{S_{n}}$	Transmission mode of *n*-th body sensor $\alpha_{S_{n}}\in \left\{0,\;1\right\}$
$\beta_{S_{n}}^{k}$	*k*-th time slot is assigned to the *n*-th body sensor for direct transmission $\beta_{S_{n}}^{k}\in \left\{0,\;1\right\},\;\left(n\in \left(1,\;2,\;\ldots ,\;N\right),\;\forall k\in \psi \right)$
$\delta_{S_{n}\to S_{m}}^{k}$	*k*-th time slot is allocated to *n*-th body sensor for transmitting data to *m*-th body sensor. $\delta_{S_{n}\to S_{m}}^{k}\in \left\{0,\;1\right\},\;\left(n,m\in \left(1,\;2,\;\ldots ,\;N\right),\;\forall k\in \psi \right)$
$\delta_{S_{m}\to H}^{k}$	*m*-th body sensor forwards the data from *n*-th body sensor to the hub at the *k*-th time slot. $\delta_{S_{m}\to H}^{k}\in \left\{0,\;1\right\}\left(n,m\in \left(1,\;2,\;\ldots ,\;N\right),\;\forall k\in \psi \right)$
$R_{n}$	Data rate of the *n*-th body sensor
${R_{n}}^{d}$	Data rate of the *n*-th body sensor in direct transmission mode
${R_{n}}^{c}$	Data rate of the *n*-th body sensor in cooperative transmission mode
$SINR_{n,k}^{d}$	SINR of *n*-th body sensor in *k*-th time slot in direct transmission mode
$SINR_{n,m,k}^{s\to r}$	SINR of the source-relay link in *k*-th time slot in cooperative transmission mode
$SINR_{n,m,k}^{r\to H}$	SINR of the relay-hub link in *k*-th time slot in cooperative transmission mode
$p_{n,k}^{d}$	Transmission power of the *n*-th body sensor in the *k*-th time slot in direct transmission mode
$g_{S_{n}\to H}$	Transmission gain between the *n*-th body sensor and hub
$p_{n,m,k}^{s\to r}$	Transmission power of *n*-th body sensor in the *k*-th time slot to *m*-th body sensor in cooperative transmission mode
$g_{S_{n}\to S_{m}}$	Transmission gain between the *n*-th body sensor and *m*-th body sensor in cooperative transmission mode
$p_{n,m,k}^{r\to H}$	Transmission power of *m*-th body sensor in the *k*-th time slot to hub in cooperative transmission mode
$g_{S_{m}\to H}$	Transmission gain between the *m*-th body sensor and hub in cooperative transmission mode
$n_{0}$	Noise power
${R_{n}}^{c,s\to r}$	Date rate of source-relay link in in cooperative transmission mode
${R_{n}}^{c,r\to H}$	Date rate of relay-hub link in in cooperative transmission mode
$DQ_{S_{n}}^{k}$	Data queue length at the *n*-th body sensor in time slot *k*
$DQ_{S_{n}}^{max}$	Maximum traffic queue length of body sensors
$A_{S_{n}}^{k-1}$	Arriving traffic packets of *n*-th body sensor in time slot *k* − 1
$EQ_{S_{n}}^{k}$	Energy queue length at the *n*-th body sensor in time slot *k*
$EQ_{S_{n}}^{max}$	Maximum energy queue length of body sensors
$E_{n,k-1}$	Amount of energy harvested by *n*-th body sensor in time slot *k* − 1
$PS_{data}$	Date packet size
$PS_{energy}$	Energy packet size
$p_{n}^{max}$	Maximum transmission power of body sensors
$EE_{S_{n}}^{k}$	Energy efficiency of *n*-th body sensor in time slot *k*

**Table 2:** The irrelevant state mapping table.

$\mathrm{State}\;\mathrm{Space}:\;\{DQ_{S_{n}}^{k},\;EQ_{S_{n}}^{k}\}$	The State Space If Needs to Be Explored
{0, 0}	No
{0, *}	No
{*, 0}	No
{*, *}	Yes

*: valid state; 0: invalid state.

**Table 3:** Computation complexity comparison between the modified and classical Q-learning algorithms.

Modified Q-Learning Algorithm	Classical Q-Learning Algorithm
*xy*	*x* ^3^ *y* ^2^
*xy*	*x* ^5^ *y* ^3^
-	-
*xy*	*x* ^(2*n*+1)^ *y* ^(*n*+1)^

**Table 4:** Simulation parameters setting.

Parameters	Value
*R*	10 m
Distance of each body sensor	Random distributed in (2, 5) m
$S_{n}$	(1:1:10)
*B*	1 MHz
$n_{0}$	−94 dBm/Hz
$p_{n}^{max}$	10 dBm
$\lambda_{t}$	(1:1:8) packet/time slot
$\lambda_{e}$	(1:1:8) packet/time slot
ψ	200
$\tau_{k}$	0.5 ms
$PS_{traffic}$	8 bits/packet
$PS_{energy}$	0.0002 J/packet
$DQ_{DUs}^{max}$	50 packets
$EQ_{DUs}^{max}$	50 packets
